# Lesions in the Posterior Visual Pathway Promote Trans-Synaptic Degeneration of Retinal Ganglion Cells

**DOI:** 10.1371/journal.pone.0097444

**Published:** 2014-05-23

**Authors:** Johannes Keller, Bernardo F. Sánchez-Dalmau, Pablo Villoslada

**Affiliations:** The Institute of Ophthalmology and Center for Neuroimmunology, Institute of Biomedical Research August Pi Sunyer (IDIBAPS) – Hospital Clínic, Barcelona, Spain; University of Düsseldorf, Germany

## Abstract

**Objective:**

Retrograde trans-synaptic degeneration of retinal ganglion cell layer (GCL) has been proposed as one of the mechanisms contributing to permanent disability after visual pathway damage. We set out to test this mechanism taking advantage of the new methods for imaging the macula with high resolution by optical coherence tomography (OCT) in patients with lesions in the posterior visual pathway. Additionally, we explored the association between thinning of GCL as an imaging marker of visual impairment such as visual field defects.

**Methods:**

Retrospective case note review of patients with retrogeniculate lesions studied by spectral domain OCT of the macula and quadrant pattern deviation (PD) of the visual fields.

**Results:**

We analysed 8 patients with either hemianopia or quadrantanopia due to brain lesions (stroke  = 5; surgery  = 2; infection  = 1). We found significant thinning of the GCL in the projecting sector of the retina mapping to the brain lesion. Second, we found strong correlation between the PD of the visual field quadrant and the corresponding macular GCL sector for the right (R = 0.792, p<0.001) and left eyes (R = 0.674, p<0.001).

**Conclusions:**

The mapping between lesions in the posterior visual pathway and their projection in the macula GCL sector corroborates retrograde trans-synaptic neuronal degeneration after brain injury as a mechanism of damage with functional consequences. This finding supports the use of GCL thickness as an imaging marker of trans-synaptic degeneration in the visual pathway after brain lesions.

## Introduction

Retrograde trans-synaptic neuronal degeneration of the retinal ganglion cells (RGCs) has been demonstrated after damage to structures in the posterior visual pathway in various settings. This phenomenon was first observed in macaques which had the striate cortex surgically excised [1], [2]. Previously thought to occur only in congenital lesions [3]–[5], it has now been demonstrated in patients with acquired posterior visual pathway lesions [6]–[8] and its time course characterised. Recently, we have provided evidence that trans-synaptic degeneration takes place in patients with MS due to lesions in optic radiations and atrophy of the visual cortex [9]. There may be a threshold for such processes to occur, as evidence of RGCs damage has not always been found, especially in cases with smaller lesions [7]. Additionally, trans-synaptic neuronal degeneration may cascade over time, increasing permanent disability long after the initial CNS damage. Although the limits to this cascading are not known, recent findings suggest that when trans-synaptic degeneration occurs in a retrograde direction, this process does not extend over more than one synapsing neuron. It appears that highly interconnecting neurons prevent extension of the damage [10]. For all these reasons, this process is a putative target for neuroprotective therapies currently under development.

Optical coherence tomography (OCT) is utilised to study the peripapillary retinal nerve fibre layer (RNFL) and the macula in optic nerve and retinal diseases [11]. Segmentation of retinal layers has allowed the study of functional areas in greater detail. This is most useful at the macula where the RGCs density is maximal and where other anatomical structures are absent. The macula may be a better location to demonstrate early damage of RGCs in ischaemic optic neuropathy and papilledema, as edema of the optic disc may mask axonal loss at the optic nerve head [11]. Direct macular RGCs thinning has also been demonstrated in patients with optic tract lesions [12], and in cases of chiasm compression. Atrophy of the ganglion cell layer (GCL) is a reliable predictor for poor visual function [13] and correlates at the topographic level with the visual fields (VF) deficits. In cases of glaucoma [14] and chiasm tumours [15], atrophy of the GCL has been shown to correlate with permanent visual field imapirment.

We hypothesised that retrograde trans-synaptic degeneration triggered by a lesion located in the posterior visual pathway will manifest with RGCs loss at the macular level. We looked for the functional implication of trans-synaptic degeneration by correlating the atrophy of the GCL and the corresponding VF defect.

## Methods

The study was approved by the IRB of the Hospital Clinic of Barcelona as part of bigger study on the damage of the visual pathway in neurological diseases. Patient records and information was anonymized and de-identified prior to analysis.

We performed a retrospective case note review of patients attending to the neuro-ophthalmology clinic at the Ophthalmology Department of the Hospital Clínic de Barcelona for evaluation of visual function after a destructive neurological injury located in the retro-geniculate portion of the visual pathway. Patients with associated ocular or neurological pathology, and with extreme refractive errors altering the OCT were excluded from the analysis. Patients were analysed at least 12 months after the CNS insult.

Full neuro-ophthalmological examination was carried out including biomicroscopy and dilated funduscopy by a senior doctor (BSD). Manifest refraction was also performed. Automated static perimetry was done with the Humphrey II analyser (Carl Zeiss Meditec, Jena, Germany) utilising the SITA Standard 24–2 strategy. Only reports with good reliability were analysed. OCT images were obtained with the Cirrus HD-OCT (Carl Zeiss Meditec, Jena, Germany) employing the macular cube (512×128 A-scans) and optic nerve cube (200×200 A-scans) acquisition protocols. Image analysis was carried out with in-built protocols.

The thickness of the supero-nasal, infero-nasal, supero-temporal and infero-temporal 60° segments of macular RGC layer was correlated with the mean pattern deviation (PD) calculated for each corresponding VF quadrant ignoring the nasal extension. Additionally, to demonstrate that the distribution of the RNFL fibres are not distributed topographically in the same way, the average for the 2 oblique clock hours representing 60° of each quadrant of the peripapillary RNFL were correlated with the same VF values. Statistical analysis was carried out using R software (R Foundation for Statistical Computing, Vienna, Austria). To study the relationship between the VF defect and the GCL thickness, correlation coefficients were calculated with the Pearson product moment method analysing right eyes separately from left eyes. Each quadrant of the visual field was correlated with its corresponding macular GCL segment and correlated with the Spearman rank correlation method. This was repeated for the RNFL. Significance level was set at 0.05.

## Results

### Clinical findings

We identified 8 patients with retrogeniculate lesions in the database of the neuro-ophthalmology clinic. Five patients had strokes, two had surgical excision of tumours and one had toxoplasma encephalitis. Four patients had right-sided lesions and four had left-sided ones. The median age (range) was 37 years (18–68, mean 40.1). The median time between the nervous system insult and the OCT scan was of 4.9 years (1.4–9.8). Their clinical features are summarised in table 1.

**Table 1:** Clinical features of patients with visual field defects secondary to retro-geniculate visual pathway lesions.

Case	Age	Sex	Site of lesion	Side	Type of lesion	Evolution (years)	Thinnest ipsilateral segment
1	49	M	Occipital cortex	Left	Intraparenchymal bleed	2.7	Infero-temporal
2	25	F	Occipital cortex	Left	Toxoplasma encephalitis	6.7	Infero-temporal
3	18	M	Occipital cortex	Left	Intraparenchymal bleed	9.8	Infero-temporal
4	68	M	Occipital cortex	Left	Ischaemic stroke	3.9	Supero-temporal
5	21	M	Occipital cortex	Right	Intraparenchymal bleed	7.1	Infero-temporal
6	54	M	Internal capsule	Right	Intraparenchymal bleed	5.8	Supero-temporal
7	65	M	Occipital cortex	Right	Post surgical excision	2.6	Supero-temporal
8	21	M	Occipital cortex	Right	Post surgical excision	1.4	Infero-temporal

**Figure 1:**
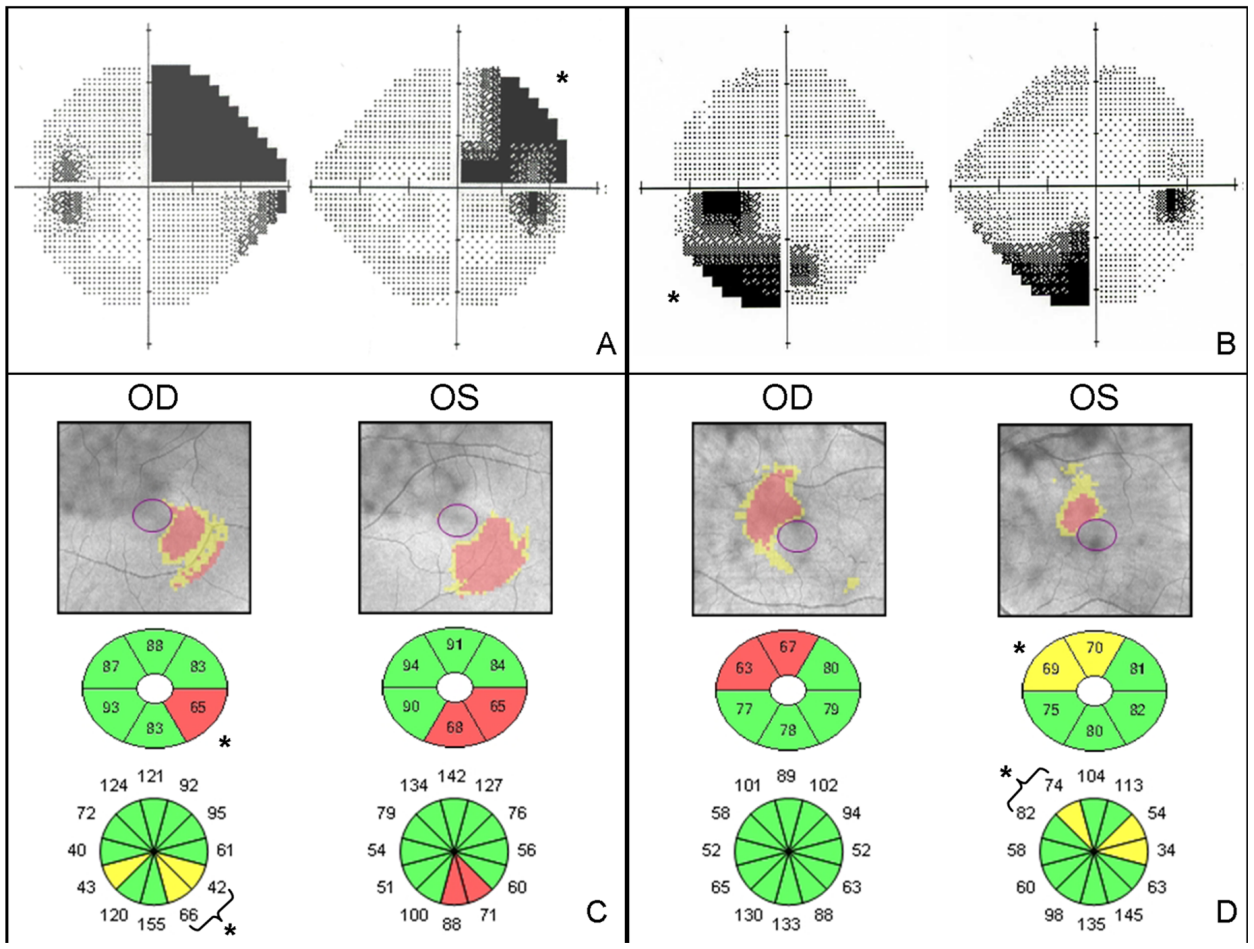
VF greyscale plot (A, B), RGC and RNFL thickness (C, D) of patient 3 (A, C) and patient 6 (B, D). * Sample correlated OCT segments with corresponding VF quadrant.

We found thinning of the GCL on the lesion's projecting sector of the macula in all cases (Figure 1 and table S1). The mean GCL thickness was 73.3 µm (SD: 7.9) OD and 74.3 µm OS (SD: 7.9). The mean thickness of the thinnest sector was 54.1 µm (SD: 8.3) OD and 59.5 µm OS (SD: 8.7).

Regarding the VF, we found mean deviation of −10.3dB (SD 6.5) in the right eye (OD) and left eye (OS) −10.4dB (SD 5.2), and PSD OD 11.7dB (SD 2.3) and OS 12.1dB (SD 2.7). There were 2 patients with homonymous hemianopia on either side while the other 6 had homonymous quadrantanopsia.

### Association of retina atrophy with visual field deficits

**Figure 2:**
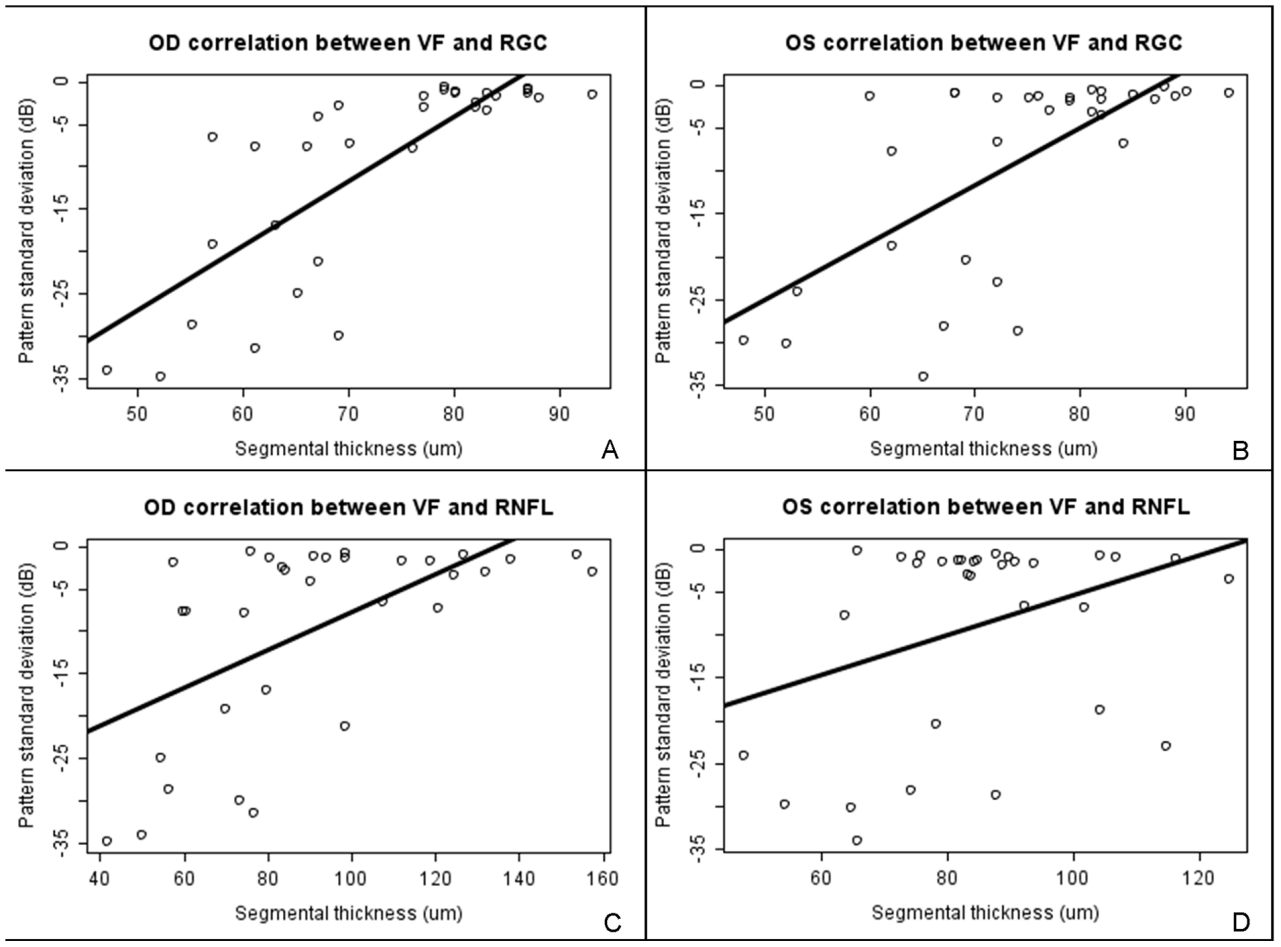
Scatterplot showing dispersion and linear regression of quadrant PD depression vs GCL thickness in the right (A) and left (B) eyes of all patients. PD depression vs RNFL thickness is shown for the right (C) and left (D) eyes.

In order to analyse the association between GCL thickness and peripapillar RNFL thickness with each quadrant of the VF, we calculated the Spearman correlation coefficient between the mean VF quadrant PD and its corresponding macular GCL segment for both eyes of each patient. We observed strong correlation between the corresponding macular segment of the GCL and the VF (Table 2). We found an overall correlation between macular GCL segment thickness and the PD of the corresponding VF quadrant of 0.792 (CI95: 0.613–0.894, p<0.001) for right eyes and 0.674 (CI95: 0.425–0.828, p<0.001) for left eyes. However, the correlation for the RNFL with the corresponding VF quadrant defect of each patient was significantly smaller (r = 0.382; 0.36 SD) (Table 2). Overall correlation between peripapillar RNFL segment thickness and the PD of the corresponding VF quadrant was 0.589 (CI95: 0.303–0.778, p<0.001) for the right eye and 0.356 (CI95: 0.009–0.627, p = 0.045) for the left eye (Figure 2).

**Table 2:** Correlation coefficients between the GCL and RNFL thickness and the VF deviation in corresponding quadrants.

	VF – RGC layer		VF – NRFL	
VF segment	r	p	r	p
**Superonasal**	0.491	<0.05	0.575	0.02
**Superotemporal**	0.712	0.002	0.605	0.013
**Inferotemporal**	0.708	0.002	0.546	0.029
**Inferonasal**	0.661	0.005	0.243	0.365

## Discussion

These findings show macular GCL atrophy in patients that have suffered a retrogeniculate lesion with at least one-year evolution. Therefore, the finding of sector macular GCL atrophy proves retrograde trans-synaptic degeneration of neurons in the visual pathway in cases without other ophthalmic or neurological disease. The functional correlation between sector thinning of the GCL and the sensitivity loss of the VF in the corresponding field indicates that this may occur in lesions regardless their size as shown by Jindahra el al. in quadrantanopic cases [16]. However, we found a significantly stronger correlation between the VF defect and the thickness of GCL rather than that of the peripapillar RNFL. This is probably due to the fact that at the macula, the bodies of the neurons are more numerous and topographically organised to correspond to the VF. In contrast, the anatomical distribution of fibres in the peripapillar RNFL is more complex, making a correlation with the visual field more difficult to establish. Another important element is the absence of blood vessels [17] or other structures that may interfere with the OCT image acquisition. The higher resolution of spectral-domain OCT also allows studying the segmentation of the retina in layers in more detail. It is noteworthy that cases with macular sparing hemi- and quadrantanopia also showed macular GCL atrophy away from the fovea, perhaps explained due to the representation of 15–20° of visual field in the area scanned by the OCT instrument used and the absence of ganglion cells on the fovea.

All patients studied presented highly correlating defects regardless the timing of their lesion [7], adding to the idea that macular changes may occur before the first year and remain stable over time. Also, patients developed RGC thinning irrespective of the type of lesion. Clinical heterogeneity may lower the correlation between the VF sensitivity and the GCL thickness if the aetiology of disease involves a diffuse process such as demyelinating disease [9], [12], multiple strokes or inflammatory encephalitis.

This study is limited from being a retrospective case series where patients had a lesion long time before presentation and with fairly heterogeneous clinical presentations. Additionally, as the inbuilt OCT protocol for the analysis of the GCL thickness was designed for glaucoma, the data cannot be analysed following the neurological topography of the eye. Development of a specific software tool for topographic analysis of the GCL following the VF projection would be of great advantage in further demarcating areas of neuronal degeneration at the macular level. Additionally, it may allow for a more precise study of congruousness of the VF defect. Longitudinal studies assessing the functional and morphological changes at the macular area derived from an acute insult to the posterior visual pathway is warranted to contribute to our understanding of visual neuronal trans-synaptic degeneration dynamics. This would also establish whether there is a threshold for RGC atrophy to become manifest.

The correlation between VF sensitivity and GCL thickness at the macula may result in a clinical application of macular OCT as a marker for the quantification of posterior visual pathway damage. This may be especially useful in patients unable to cooperate with functional testing. It may also serve as an additional measure to demarcate VF defects and as predictor of a permanent vision defect. Finally, current research in development of new neuroprotective therapies will require imaging markers of axonal degeneration as surrogate outcomes in clinical trials. Thinning of macular GCL after retrogeniculate lesions may provide one such measurement.

## Supporting Information

Table S1 **Clinical, OCT and VF raw data from patients.**
(XLSX)
